# A selective and mild glycosylation method of natural phenolic alcohols

**DOI:** 10.3762/bjoc.12.51

**Published:** 2016-03-15

**Authors:** Mária Mastihubová, Monika Poláková

**Affiliations:** 1 Institute of Chemistry, Slovak Academy of Sciences, Dúbravská cesta 9, SK-845 38 Bratislava, Slovakia

**Keywords:** diastereoselectivity, *p*-hydroxyphenylalkyl glycosides, mild promoters, natural products, 1,2-*trans*-glycosylation

## Abstract

Several bioactive natural *p*-hydroxyphenylalkyl β-D-glucopyranosides, such as vanillyl β-D-glucopyranoside, salidroside and isoconiferin, and their glycosyl analogues were prepared by a simple reaction sequence. The highly efficient synthetic approach was achieved by utilizing acetylated glycosyl bromides as well as aromatic moieties and mild glycosylation promoters. The aglycones, *p*-*O*-acetylated arylalkyl alcohols, were prepared by the reduction of the corresponding acetylated aldehydes or acids. Various stereoselective 1,2*-trans-O-*glycosylation methods were studied, including the DDQ–iodine or ZnO–ZnCl_2_ catalyst combination. Among them, ZnO–iodine has been identified as a new glycosylation promoter and successfully applied to the stereoselective glycoside synthesis. The final products were obtained by conventional Zemplén deacetylation.

## Introduction

**Figure 1:**
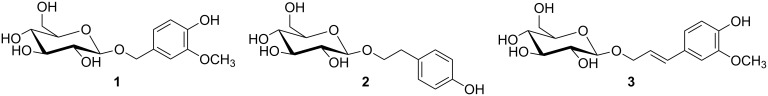
Structures of vanillyl β-D-glucoside (**1**), salidroside (**2**) and isoconiferin (**3**).

Arylalkyl (substituted benzyl, phenethyl and phenylpropenyl) glycosides having a free phenolic function at the *para*-position of the aglycone are substances widely occurring in plants. They exhibit numerous biological activities which are in many cases related to their structure–antioxidant activity relationship. 4-Hydroxy-3-methoxybenzyl β-D-glucopyranoside (vanillyl β-D-glucoside, **1**) isolated from the exocarp of *Juglans mandshurica* Maxim showed antibacterial activity [1]. A wide range of pharmacological effects, e.g., anti-oxidant, anti-inflammatory, anticancer, hepatoprotective, cardioprotective, neuroprotective, antidiabetic, and antiviral activities [2–13] are reported for 4-hydroxyphenethyl β-D-glucopyranoside (salidroside, **2**) which is known to be the main bioactive component of plants of the genus *Rhodiola*. 4-Hydroxy-3-methoxycinnamyl β-D-glucopyranoside (isoconiferin or citrusin D, **3**) has been shown to exhibit a hypotensive effect [14] (Figure 1).

The activities of the above mentioned glycosides are primarily related to the structure of the aglycone. The glycosylation of the poorly soluble hydroxyalkylphenols, such as 4-hydroxybenzyl, vanillyl, 4-hydroxyphenethyl and coniferyl alcohols, significantly increases their water solubility. Further it influences the physicochemical and pharmacological properties of these phenols and often reduces their potential toxicity. Moreover, *p*-hydroxyarylalkyl glycosides are also key starting building blocks for the synthesis of more complex bioactive natural compounds with promising therapeutic potential (e.g., phenylpropanoid glycosides) [15–16]. Therefore, the development of relatively simple and safe procedures is needed for a rapid multigram-scale synthesis of arylalkyl glycosides in good yields.

The total syntheses of compounds **1**, **2** and **3** and their structurally related glycosides employing various chemical [9,17–23] or enzymatic [24–29] methods have been previously reported. The most frequently used protocol under Koenigs–Knorr conditions is represented by the reaction of an acetobromoglucose and (4-*O*-benzyloxyphenyl)alkyl alcohol catalysed by Ag salts [9,18]. The final removal of the benzyl protecting group from the phenolic function of the aglycone by catalytic reduction can be however problematic in the case of more complex molecules containing for example double bonds (e.g., arenarioside) [30]. Isoconiferin (**3**) has been prepared mainly by the trichloroacetimidate method [20–21] using 4-*O*-acetylated coniferyl alcohol as the acceptor. On the other hand, the Mizoroki–Heck reaction of 4-hydroxy-3-methoxyphenylboronic acid and peracetylated allyl β-D-glucoside has been used to synthesize **3** in 52% yield [22].

Enzymatic glycosylations of arylalkyl alcohols are easily accomplished, however, the glycosides have often been obtained in low to moderate yields (usually below 30%). Glucosidases from fruit seed meals are the most commonly used biocatalysts for reverse hydrolysis reactions carried out in organic media or ionic liquids as co-solvents [24–27]. The enzymatic transglucosylation using 4-nitrophenyl β-D-glucopyranoside as the donor and almond β-glucosidase as biocatalyst gave salidroside (**2**) in moderate yield [28].

Recently, we have published the enzymatic glycosylation of tyrosol (2-(4-hydroxyphenyl)ethanol) with cellobiose, lactose and melibiose as donors for the preparation of salidroside and its α- and β-galactoside analogues [31]. However, all transglycosylation reactions required a distinct pair of the disaccharide donor and the glycosidase for which the reaction conditions had to be optimized. The current paper deals with an efficient, safe and uniform chemical synthesis of various *p*-hydroxyarylalkyl glycosides, including compounds **1**–**3**.

## Results and Discussion

### Preparation of aglycones

**Scheme 1:**
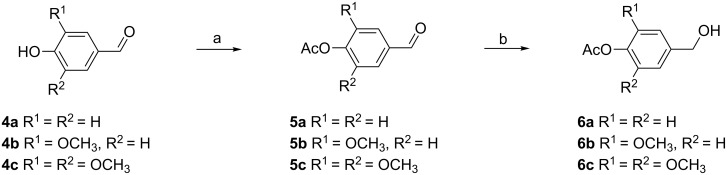
Reagents and conditions: a) Ac_2_O, pyridine, rt, 10 h, >98%; b) NaBH_4_, H_3_PO_4_, −5 °C, 85–95%.

The synthesis of the appropriate aglycones **6a–c** was commenced from readily available commercial *p*-hydroxyphenylcarbaldehydes **4a**–**c** which are less expensive than the corresponding *p*-hydroxybenzyl alcohols (Scheme 1). The conventional acetylation of **4a–c** with acetic anhydride in pyridine gave *p*-acetoxyphenylcarbaldehydes **5a–c** in more than 98% yields. Subsequently the aldehyde function was reduced by NaBH_4_ at pH 7–8, which was kept constant by the continuous addition of 85% H_3_PO_4_ to avoid phenolic acetyl-group cleavage. The *p*-acetoxybenzyl alcohols **6a–c** were isolated in 85–95% yields.

**Scheme 2:**
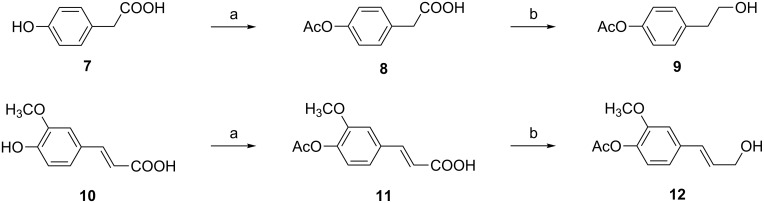
Reagents and conditions: a) Ac_2_O, H_2_SO_4_, 5 °C to rt, 30 min, >94%; b) 1. NaBH_4_, THF, 5 °C, 10 min, 2. I_2_, 5 °C, 15 min, rt, 3 h, 84% for **9**, 69% for **12**.

*p*-Hydroxyphenylacetic acid (**7**) and ferulic acid (**10**) are also more readily available on the market than the corresponding alcohols, tyrosol and coniferyl alcohol. Therefore *p*-*O*-acetylated tyrosol (**9**) and *p*-*O*-acetylated coniferyl alcohol (**12**) were prepared from acids **7** and **10** by a two-step sequence: acid-catalysed acetylation of the phenolic hydroxy group (isolated yields >94%) followed by the reduction of the carboxylic function with NaBH_4_–I_2_ in THF (Scheme 2). Due to the higher lability of the phenolic acetate under basic conditions, the method published by Kanth and Periasamy [32] was slightly modified. For this, the reagents were added at lower temperature, the reaction time was prolonged and a solution of NaHCO_3_ instead of NaOH was used for washing. Under these conditions, the deacetylated product was formed only in traces (<5%). Regarding the reduction of acetylated ferulic acid **11**, no formation of the 1,4-reduction product was observed and the double bond remained untouched.

### Glycosylation reactions

Acetyl-protecting groups are the simplest choice also for the protection of the glycone part since the deprotection of both, sugar and aromatic moieties, can be accomplished in one step. Naturally occurring *O*-glycosides possess mostly 1,2-*trans-*glycosidic linkages. Therefore, neighbouring group participation is usually exploited in the *trans*-*O*-glycosylation of appropriate aglycones.

In the course of our synthetic studies, 1,2-*trans*-glycosylation reactions utilizing per-*O*-acetyl-D-glucopyranose as a donor were initially investigated. However, the reaction of **6b** with per-*O*-acetylated-D-glucopyranose promoted by a Lewis acid (SnCl_4_) in DCM failed. The deacetylated aglycone – vanillyl alcohol along with some amounts of 2,3,4,6-tetra-*O*-acetyl-D-glucopyranose were isolated. It is evident that these frequently used reaction conditions require more acid-stable derivatives. Therefore, it was reasonable to look for milder conditions for an efficient and inexpensive method of glycosylation while excluding the use of toxic mercury salts as promoter (Helferich reaction) or silver salts. The latter are often rather expensive, moisture and light sensitive, and uncomfortable to handle.

**Figure 2:**
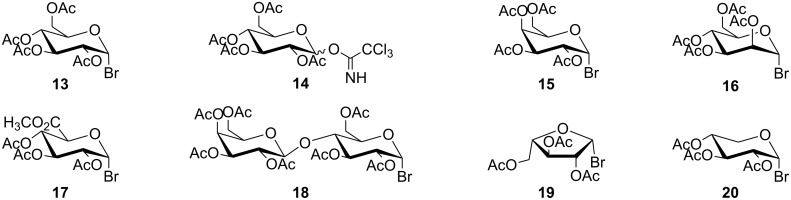
Synthesized glycosyl donors.

Accordingly, various acetylated glycosyl bromides **13**, **15**–**20** derived from pyranoses, furanoses and a disaccharide (Figure 2) were prepared as glycosyl donors in one step and high yields starting from the peracetylated sugars.

**Scheme 3:**
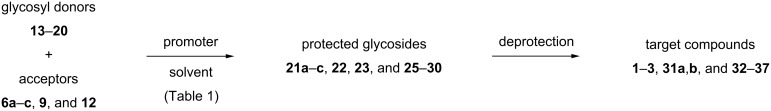
General reaction scheme for the synthesis of *p*-hydroxyphenylalkyl glycosides.

The glycosyl bromides depicted in Figure 2 were subsequently examined in the glycosylation of acceptors **6a–c**, **9** and **12** (Scheme 3, Table 1). The choice of the glycosylation promoter was strongly limited by the instability of the phenolic acetyl group under basic as well as strongly acidic conditions. Only mild, neutral promoters were therefore selected and investigated. Thus, the reactions were performed in the presence of known mild catalysts such as Ag_2_O [33] (method A), the less frequently used ZnO–ZnCl_2_ system [34] (method B) and the combination of 2,3-dichloro-5,6-dicyano-1,4-benzoquinone (DDQ) with iodine (DDQ–I_2)_ [35] (method C). In addition ZnO–I_2_ (method D) was successfully applied as a new promoter in the stereoselective 1,2-*trans*-glycoside synthesis (Table 1, entries 4, 9, 13, 17, 19 and 25). The selection of methods C and D was based on the common knowledge that iodine, either alone or in combination with other promoters such as salts of various metals (other than the traditional Koenigs–Knorr heavy metals), serves as an effective activator of disarmed glycosyl halides in the 1,2-*trans*-glycoside synthesis [35–38]. Despite the fact that the precise mechanism is not clear, it is assumed that the reaction of the glycosyl bromide promoted by iodine (through the formation of an iodobromonium ion) results in a carbohydrate-derived oxocarbonium ion that functions as the reactive intermediate [35].

**Table 1:** Synthesis of **21a–c** to **30** under various conditions.

Entry	Donor	Acceptor	Method^a^	Mol. sieves	Temp.	Time (min)	Product	Yield (%)	α:β^b^

1	**13**	**6b**	A	No	rt	180	**21b**	57	0:1
2	**13**	**6b**	B	Yes	rt	90	**21b**	46	0:1
3	**13**	**6b**	C	Yes	rt	40	**21b**	68	0:1
4	**13**	**6b**	D	Yes	rt	360	**21b**	56	0:1
5	**13**	**6a**	C	Yes	rt	20	**21a**	78	0:1
6	**13**	**6c**	C	Yes	rt	60	**21c**	63	0:1
7	**13**	**9**	B	Yes	rt	90	**22**	49	0:1
8	**13**	**9**	C	Yes	rt	110	**22**	61	0:1
9	**13**	**9**	D	Yes	rt	360	**22**	63	0:1
10	**13**	**12**	C	Yes	rt	15	**23** **24**	<5 58	n.d. 0:1
11	**14**	**12**	E	Yes	–^d^	30	**23**	55	0:1
12	**15**	**6b**	C	Yes	rt	20	**25**	68	0:1
13	**15**	**6b**	D	Yes	rt	360	**25**	63	0:1
14	**16**	**6b**	C	Yes	rt	30	**26**	70	1:0
15	**17**	**6b**	C	Yes	rt	30	**27**	70	0:1
16	**18**	**6b**	C	Yes	rt	30	**28**	50	0:1
17	**18**	**6b**	D	Yes	rt	360	**28**	46	0:1
18	**19**	**6b**	C	Yes	rt	20	**29**	66	1:0
19	**19**	**6b**	D	Yes	rt	45	**29**	62	1:0
20	**20**	**6b**	C	Yes	rt	20	**30**α/β	56	1:2.3
21	**20**	**6b**	C	No	rt	25	**30**α/β	52	1:4
22	**20**	**6b**	C^c^	No	rt	25	**30**α/β	45	1:2.1
23	**20**	**6b**	C	No	4 °C	30	**30**α/β	78	1:4.7
24	**20**	**6b**	B	No	4 °C	90	**30**α/β	50	1:3.5
25	**20**	**6b**	D	No	4 °C	75	**30**β	72	0:1

^a^Method: A) Ag_2_O; B) ZnO–ZnCl_2_; C) DDQ–I_2_; D) ZnO–I_2_; E) TMSOTf. ^b^Anomeric ratios were determined by integration of the appropriate peaks in the ^1^H NMR spectra; n.d. – not determined. ^c^DCM was used as the solvent. ^d^Temperature −78 to 0 °C.

In the first step, acceptor **6b** and glucopyranosyl bromide **13** as the donor were selected and tested in the presence of the above mentioned promoters (see Table 1, entries 1–4) in order to identify the optimal glycosylation conditions in terms of yield and selectivity. In all cases, only the 1,2-*trans*-glycosylation product, β-glucoside **21b**, was obtained. While method B (ZnO–ZnCl_2_) performed in DCM instead of ACN afforded only a moderate yield (Table 1, entry 2, 46%) of **21b**, the reactions in DCM promoted by Ag_2_O (Table 1, entry 1, 57%) and ZnO–I_2_ (Table 1, entry 4, 56%) gave comparably good yields. DDQ–I_2_ in ACN (Table 1, entry 3, 68%) gave **21b** in the highest yield, in addition to the exclusive selectivity and the shortest reaction time (Table 1, entry 5). Therefore this promoter was selected for the glucosylation reactions of acceptors **6a** and **6c** with bromide **13**, affording compounds **21a** and **21c** with full β-selectivity.

Acetylated tyrosol **9** as glycosyl acceptor reacted only smoothly with **13** under all three studied reaction conditions (Table 1, entries 7–9) affording the acetylated salidroside **22** from moderate (ZnO–ZnCl_2_, 49%) to good yields (DDQ–I_2_, 61% and ZnO–I_2_, 63%) with strict β-stereocontrol.

On the other side, the glucosylation of *p*-*O*-acetylated coniferyl alcohol **12** with bromide **13** failed under these conditions. Coniferyl aldehyde **24** was detected and isolated as a major product. For example, the DDQ–I_2_-promoted reaction provided aldehyde **24** in 58% yield along with less than 5% of the desired product **23**. This may be caused by the oxidative nature of the promoter and by the existence of a conjugated electronic push–pull system of coniferyl alcohol that is enhanced by the electron-withdrawing acetoxy group. On the contrary, the TMSOTf-promoted glycosylation [39] (method E) of coniferyl alcohol **12** with trichloroacetimidate **14** at low temperature was found to be more efficient and glycoside **23** was obtained in high yield with full β-selectivity as proved by NMR spectroscopy. The phenolic acetyl group remained intact under these conditions.

Six distinct acetylated glycosyl donors **15**–**20** were further examined to prove the feasibility of the method. D-Galacto-, D-manno- and methyl D-glucuronate-derived donors **15**–**17** originated from hexopyranoses. The pentosyl donors, which usually exhibit a diminished glycosylation selectivity, were represented by L-arabinofuranosyl bromide **19** and D-xylopyranosyl bromide **20**.

The glycosylation of **6b** with galactosyl, glucuronic acid methyl ester or lactosyl bromides **15**, **17** and **18**, proceeded also stereoselectively and the β-anomers of glycosides **25**, **27** and **28** were isolated in good yields (50–68%) as the only products. The structures of all β-anomers (**21a–c**, **22**, **23**, **25**, **27** and **28**) were confirmed by the presence of a doublet of the anomeric proton with characteristic vicinal interaction constant ^3^*J*_1,2_ in the interval of 7.5–7.9 Hz in the ^1^H NMR spectra. The glycosidic bond between galactose and glucose in lactosyl bromide **15** was not affected under the examined conditions. On the other hand, the reaction of D-mannosyl and L-arabinosyl bromides **16** and **19** with **6b** afforded solely α-anomers **26** and **29** as the 1,2-*trans*-glycosylation products. The α-manno-configuration of glycoside **26** was proven by the characteristic coupling constant (^1^*J*_C1,H1_ = 170.3 Hz). The α-configuration of the L-arabinofuranoside **29** was confirmed by ^1^H NMR (broad singlet at 5.09 ppm) and ^13^C NMR spectra (C-1 at 104.6 ppm).

In contrast to the above mentioned results, the reaction of D-xylosyl bromide **20** with **6b** did not proceed stereoselectively. An anomeric mixture of vanillyl xylosides **30**α/β in a ratio varying from 1:2.3 to 1:4 was obtained when the glycosylation was promoted with DDQ–I_2_ in either DCM or ACN at room temperature (Table 1, entries 20–22). The same reaction performed at 4 °C provided again a mixture of xylosides **30**α/β (Table 1, entry 23) but with a slightly higher selectivity (**30**α/β in ratio 1:4.7). Therefore, to improve the selectivity, other promoters were also examined at low temperature. The use of ZnO–ZnCl_2_ in DCM (Table 1, entry 24) led again to a mixture of xylosides **30**α/β. In contrast, the glycosylation promoted by ZnO–I_2_ in DCM (Table 1, entry 25) was the only condition affording **30** as pure β-anomer (72%). The lack of selectivity at room temperature can be explained by a higher thermodynamic stability of the α-xylopyranosides compared to the corresponding β-xylopyranosides. Apparently, anomerisation of the kinetically formed β-anomer easily takes place under mild reaction conditions at ambient temperature [40], but it is suppressed by decreased temperature in combination with an appropriate promoter (ZnO–I_2_). Moreover, this new promoter was successfully applied to the reactions of the corresponding acceptor and four other glycosyl bromides, i.e., D-glucosyl (Table 1, entries 4 and 9), D-galactosyl (Table 1, entry 13), lactosyl (Table 1, entry 17) and L-arabinosyl (Table 1, entry 19). The glycosylation reactions of the latter donors catalysed by ZnO–I_2_ gave comparable yields and were completely stereoselective similarly to other promoters, although they required at least a 2-times longer reaction time.

**Figure 3:**
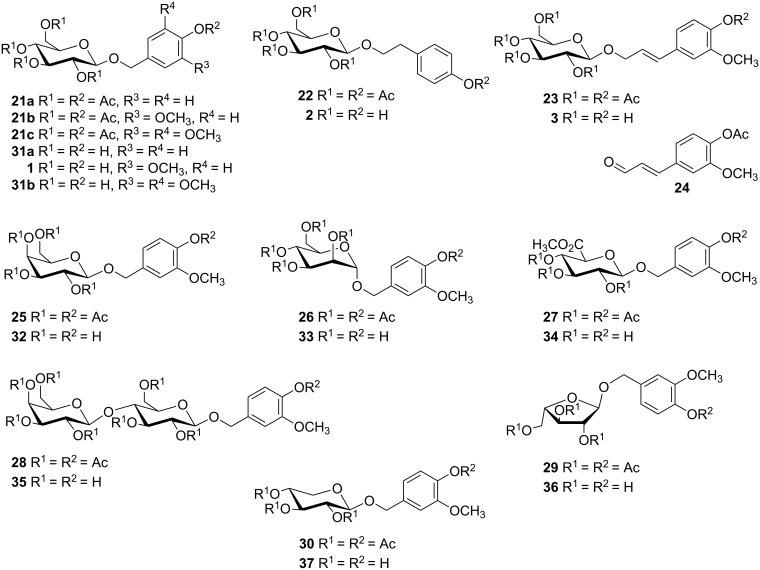
Overview of protected and deprotected products.

In the final step, the removal of the acetyl groups under Zemplén conditions proceeded smoothly and the desired target glycosides **1–3**, **31a**,**b** and **32–37** were isolated in high yields (Figure 3).

## Conclusion

The glycosylation methods studied in this work represent a simple and convenient approach to bioactive natural *p*-hydroxyphenylalkyl glycosides and their analogues. The mild reaction conditions with exclusive stereoselectivity can be used as an alternative to the common Koenigs–Knorr or Helferich glycosylation. In many cases, the DDQ–I_2_-promoted reaction provided products in a stereoselective way and in the highest yields. It is noteworthy that ZnO–I_2_ is a new glycosylation promoter, which was found to well activate also less reactive disarmed tetra-*O*-acetyl-α-D-glycopyranosyl bromides, to give stereoselectively only the 1,2-*trans* glycosides in good to high yields. These conditions were efficiently used in the stereoselective xyloside synthesis that is not trivial. All used glycosylation conditions were compatible with acetyl protective groups of the phenolic function. The coupling reaction and deprotection were achieved in two steps, thus providing the rapid access to the targeted glycosides.

## Supporting Information

File 1 Experimental procedures and analytical data.
