# Supplementary material for: A selective and mild glycosylation method of natural phenolic alcohols
Source: Beilstein J Org Chem. 2016 Mar 15;12:524–30. doi: 10.3762/bjoc.12.51 (PMC4901888; doi:10.3762/bjoc.12.51)
Supplement: File 1 — Experimental procedures and analytical data. [file Beilstein_J_Org_Chem-12-524-s001.pdf]

Supporting Information

for

A selective and mild glycosylation method of natural phenolic alcohols

Mária Mastihubová* and Monika Poláková

Address: [1]Institute of Chemistry, Slovak Academy of Sciences, Dúbravská cesta 9, SK-845 38 Bratislava, Slovakia

Email: Mária Mastihubová - chemjama@savba.sk

* Corresponding author

Experimental procedures and analytical data

Experimental

General: The reactions were performed with commercial reagents purchased from Sigma-Aldrich, Acrōs Organics, or Merck. Flash column chromatography was carried out on silica gel 60 (0.040–0.060 mm, Merck or 0.035–0.075 mm, Acrōs Organics) using distilled solvents (toluene, ethylacetate, chloroform, methanol). Dichloromethane and acetonitrile were dried (CaH_2) and distilled before use. All reactions using sensitive reagents were carried out under an argon atmosphere. Molecular sieves (4 Å) were microwave-dried before use. TLC was performed on aluminium sheets precoated with silica gel 60 F_{254} (Merck). Spots were visualized by UV light (λ_{max} = 254 nm) and charred with 5% sulfuric acid in ethanol (1% orcinol). Melting points were recorded with a Kofler hot block and are uncorrected. ^{1}H NMR and ^{13}C NMR spectra were recorded at 25 °C with a 300 MHz Bruker AM 300 spectrometer, 400 MHz Varian 400 NMR spectrometer or a 400 MHz Bruker AVANCE III HD 400 MHz equipped with a Prodigy CryoProbe. Chemical shifts are referenced to either TMS (δ 0.00, $CDCl_3$ for ^{1}H) or HOD (δ 4.79, CD_3OD for ^{1}H), and to internal $CDCl_3$ (δ 77.16, ^{13}C) or CD_3OD (δ 49.00, ^{13}C). Optical rotations were measured on Perkin–Elmer 241 and Jasco P2000 polarimeters at 20 °C. High resolution mass spectrometry was performed on a Premier Q-TOF mass spectrometer (Waters) or an Orbitrap Velos PRO spectrometer (Thermo Fisher Scientific).

General procedure for the preparation of 6a–c: To a stirred solution of aldehyde **4a–c** (40 mmol) in dry pyridine (25 mL) acetic anhydride (25 mL) was added at 0 °C. The mixture was then stirred at rt for 10 h, then diluted with ice-cold water (200 mL), and stirred for another 2 h. The aqueous layer was extracted with EtOAc (2 x 100 mL), the combined organic phase was washed with water (3 x 50 mL), dried (Na_2SO_4) and concentrated. The conversions of the acetylation reactions were at least 98%. The crude acetylated aldehyde **5a–c** (20 mmol) was dissolved in MeOH (50 mL) and cooled to −5 °C. Under stirring, $NaBH_4$ (1.2 equiv) and 85% H_3PO_4 (0.4 equiv) were added in three portions and the progress of the reduction was monitored by TLC ($CHCl_3$/MeOH 9.5:1). When the reaction was complete, the pH of the

reaction mixture was adjusted to pH 5 using 85% H_3PO_4, salts were removed by filtration and the solvent was evaporated. The residue was dissolved in EtOAc (50 mL), washed with water (3 x 50 mL), brine (50 mL), dried (Na_2SO_4) and concentrated. Purification by column chromatography ($CHCl_3$/MeOH 1:0 $\rightarrow$9.5:1) gave the desired alcohols **6a–6c**.

4-Acetoxybenzyl alcohol (6a): Colourless oil, yield 95%. ^{1}H NMR (300 MHz, $CDCl_3$): δ = 2.30 (s, 3 H, $OCOCH_3$), 4.67 (s, 2 H, CH_2), 7.08 (d, 2 H, J = 8.5 Hz, H-arom), 7.37 (d, 2 H, J = 8.0 Hz, H-arom). ^{13}C NMR data are in agreement with literature [1].

4-Acetoxy-3-methoxybenzyl alcohol (6b): Colourless oily solid, yield 89%. ^{1}H NMR (300 MHz, $CDCl_3$): δ = 2.31 (s, 3 H, $OCOCH_3$), 3.84 (s, 3 H, OCH_3), 4.67 (s, 2 H, CH_2), 6.92 (dd, J = 2 Hz, J = 8 Hz, 2 H, H-arom), 7.01 (d, J = 8 Hz, 2 H, H-arom). ^{13}C NMR (101 MHz, $CDCl_3$): δ = 20.6 (OCOCH$_3$), 55.8 (OCH_3), 65.0 (CH_2), 111.0, 118.9, 122.6 (CH-arom), 139.0, 139.9, 151.1 (C-arom), 169.2 (OCOCH$_3$).

4-Acetoxy-3,5-dimethoxybenzyl alcohol (6c): A pale yellow solid, yield 85%, m. p. 102-104 ºC, 93.5-95.5 ºC [2]. ^{1}H NMR (300 MHz, $CDCl_3$): δ = 2.34 (s, 3 H, $OCOCH_3$), 3.82 (s, 6 H, 2x OCH_3), 4.65 (s, 2 H, CH_2), 6.63 (s, 2 H, H-arom). ^{13}C NMR (101 MHz, $CDCl_3$): δ = 20.4 (OCOCH$_3$), 56.1 (OCH_3), 65.3 (CH_2), 103.2(2x) (CH-arom), 127.8, 139.4, 152.1 (4x C-arom), 168.8 (OCOCH$_3$).

General procedure for preparation of 9 and 12: Acid **7** or **10** (50 mmol) was suspended in acetic anhydride (15 mL) and 2 drops of conc. H_2SO_4 were added at 5 °C. The mixture was stirred for 30 min at rt, then diluted with ice-cold water (200 mL), and stirred for additional 2 h. The aqueous layer was extracted with EtOAc (2 x 150 mL), the combined organic phase was washed with water (3 x 50 mL), dried (Na_2SO_4) and concentrated. Conversions for acetylation reactions were at least 94%. The crude acetylated acids were crystalized from aqueous ethanol. The crude acetylated carboxylic acid **8** or **11** (20 mmol) was dissolved in THF (80 mL) and $NaBH_4$ (0.9 g, 24 mmol) was added in portions at 5 °C. The reaction mixture was stirred until gas evolution ceased. Next, iodine (2.54 g, 10 mmol) dissolved in

THF (20 mL) was added dropwise into the stirred mixture over 15 min at 5 °C. Additional H_2 gas evolved and the red colour of iodine disappeared. The reaction mixture was stirred for 3 h at rt, then 3 M HCl (10 mL) was carefully added at 5 °C. Tetrahydrofuran was removed by evaporation and the mixture was extracted with diethyl ether (3 x 20 mL). The ethereal layer was washed with saturated $NaHCO_3$ (20 mL), $Na_2S_2O_3$ (20 mL) and brine (2 x 20 mL), dried (Na_2SO_4) and the solvent was evaporated. TLC ($CHCl_3$/MeOH 9:1) showed that the residues still contained traces of the deacetylated products. Column chromatography on silica gel (toluene/EtOAc 3:1) afforded **9** or **12**, respectively.

2-(4-Acetoxyphenyl)ethanol (9): Colourless oil, yield 84%. ^{1}H NMR (400 MHz, $CDCl_3$): δ = 2.28 (s, 3 H, $OCOCH_3$), 2.83 (t, 2 H, *J* = 6.6 Hz, CH_2), 3.80 (t, 2 H, *J* = 6.3 Hz, CH_2), 7.01 (d, 2 H, *J* = 8.5 Hz, H-arom), 7.22 (d, 2 H, *J* = 8.4 Hz, H-arom). ^{13}C NMR (101 MHz, $CDCl_3$): δ = 21.0 (OCO*C*H_3), 38.5 (CH_2), 63.4 (CH_2), 121.5 (CH-arom), 129.9 (CH-arom), 136.2 (C-arom), 149.1 (C-arom), 169.7 (O*C*OCH_3).

(*E*)-3-(4-Acetoxy-3-methoxyphenyl)-2-propen-1-ol (12): Pale yellow oil, yield 69%. ^{1}H NMR (300 MHz, $CDCl_3$): δ = 2.31 (s, 3 H, $OCOCH_3$), 3.84 (s, 3 H, OCH_3), 4.32 (dd, 2 H, *J* = 1.4, 5.6 Hz, CH_2), 6.31 (dt, 1 H, *J* = 5.6, 15.9 Hz, H-A), 6.58 (d, 1 H, *J* = 15.9 Hz, H-B), 6.92-7.00 (*m*, 3 H, H-arom). ^{13}C NMR (75 MHz, $CDCl_3$) δ = 20.7 (OCO*C*H_3), 55.8 (OCH_3), 63.2 (CH_2), 110.1, 119.0, 122.7 (CH-arom), 129.1 (CH-A), 135.9 (CH-B), 130.0, 139.1, 150.1 (C-arom), 169.3 (O*C*OCH_3).

General procedures for the syntheses of donors: Glycosyl bromides **13**, **15**, **16**, **18** and **20** were prepared from the corresponding peracetylated sugars according to previously reported procedures [3]. 2,3,4,6-Tetra-*O*-acetyl-1-*O*-trichloroacetimidoyl-β-D-glucopyranoside (**14**) [4], methyl 2,3,4-tri-O-acetyl-α-D-glucopyranosyluronate bromide (**17**) [5] and acetylated L-arabinofuranosyl bromide **19** [6] were prepared according to published methods.

General procedures for glycosylation reactions

Method A, promoter Ag_2O: 2,3,4,6-Tetra-*O*-acetyl-D-glucopyranosyl bromide (**13**, 0.41 g, 1.0 mmol), (4-acetoxy-3-methoxy)benzyl alcohol (**6b**, 0.22 g, 1.15 mmol) and activated 4 Å molecular sieves (1.0 g) were stirred in dry DCM (20 mL) under argon atmosphere in the dark for 30 minutes. Then the mixture was cooled to 0 °C, Ag_2O (0.46 g, 2.0 mmol) was added and stirring was continued for 2 h at rt. The solution was diluted with DCM (100 mL), filtered through a pad of Celite, the organic phase washed with water (2 x 50 mL), dried (Na_2SO_4) and concentrated. Purification of the residue by column chromatography (toluene:EtOAc 1:0 $\rightarrow$ 3:1) gave **21b**.

Method B, promoter ZnO–$ZnCl_2$: Glycosyl bromide (1.0 mmol) and the acetylated arylalkyl alcohol (1.15 mmol) were dissolved in dry DCM (10 mL) containing activated 4 Å molecular sieves (0.8 g). ZnO (1.2 mmol) and $ZnCl_2$ (1.2 mmol) were added to the stirred solution and the reaction mixture was stirred under an Ar atmosphere at rt for 1.5 h. Then the mixture was filtered and the solid residue was washed with DCM (100 mL). The organic filtrates were combined, washed with satd $NaHCO_3$ (30 mL) and brine (2 x 50 mL), dried (Na_2SO_4) and concentrated. The crude product was purified by column chromatography (toluene/EtOAc 1:0 $\rightarrow$3:1).

Method C, promoter DDQ–I_2: Glycosyl bromide (1.0 mmol), acetylated arylalkyl alcohol (1.15 mmol), activated 4 Å molecular sieves (0.4 g, optional), were stirred in dry CH_3CN (10 mL), or optionally DCM (10 mL) under argon atmosphere. To this solution, DDQ (0.25 mmol) and I_2 (0.5 mmol) were added. When TLC (toluene/EtOAc 1:1) indicated the completion of the reaction, the reaction mixture was diluted with $CHCl_3$ (100 mL), molecular sieves were filtered off, saturated $Na_2S_2O_3$ (40mL) was added and the mixture was stirred for 30 min. The layers were separated, the organic phase was washed with brine (2 x 50mL), dried (Na_2SO_4) and concentrated. The crude product was purified by column chromatography (toluene/EtOAc 1:0$\rightarrow$6:1).

Method D, promoter ZnO–I_2: Glycosyl bromide (1.0 mmol) and acetylated arylalkyl alcohol (1.15 mmol) were dissolved in dry DCM (10 mL), optionally containing activated 4 Å molecular sieves (0.8 g), and ZnO (2.4 mmol) and I_2 (1.0 mmol) were added. The reaction mixture was then stirred under argon atmosphere at rt until completion of the reaction (TLC). Then the reaction mixture was filtered, saturated $Na_2S_2O_3$ (50 mL) was added and the mixture was stirred until decolorisation. Afterwards the organic phase was separated, washed with saturated $NaHCO_3$ (30 mL) and brine (2 x 50 mL), dried (Na_2SO_4) and concentrated. The crude product was purified by column chromatography (toluene/EtOAc 1:0 $\rightarrow$ 3:1).

Method E, promoter TMSOTf: Trichloroacetimidate **14** (1.0 mmol) and acceptor **12** (1.2 mmol) were dissolved in DCM (6 mL) and activated 4 Å molecular sieves (0.5 g) were added. The mixture was stirred for 30 min at −20 °C under argon atmosphere, then cooled to −78 °C and TMSOTf (cat., 10 μL) was added and the stirring was continued for further 30 min after which the temperature was brought up to 0 °C. The reaction mixture was neutralized by addition of Et_3N and concentrated. The crude product was purified by column chromatography (toluene/EtOAc 5:1 $\rightarrow$ 3.5:1) to give **23**.

4-Acetoxybenzyl 2,3,4,6-tetra-*O*-acetyl-β-D-glucopyranoside (21a): White crystals, m. p. 101-102 ºC (*i*-propanol), $[\alpha]_D$ - 38.2 (*c* 1.0, $CHCl_3$). ^1H NMR (300 MHz, $CDCl_3$): δ = 2.01, 2.02, 2.04, 2.10, 2.30 (each s, 15 H, 5x $OCOCH_3$), 3.69 (ddd, 1 H, *J* = 2.5, 4.6, 9.5 Hz, H-5), 4.18 (dd, 1 H, *J* = 2.4, 12.3 Hz, H-6a), 4.28 (dd, 1 H, *J* = 4.7, 12.3 Hz, H-6b), 4.57 (d, 1 H, *J* = 7.8 Hz, H-1), 4.62 and 4.88 (each d, 2x 1 H, *J* = 12.4 Hz, CH_2), 5.04-5.19 (m, 3 H, H-2, H-3, H-4), 7.07 (d, 2 H, *J* = 8.5 Hz, H-arom), 7.30 (d, 2 H, *J* = 8.5 Hz, H-arom). ^{13}C NMR (75 MHz, $CDCl_3$): δ = 20.6, 21.1 (5x OCO*C*H_3 overlap), 61.9 (C-6), 68.4 (CH_2), 70.1, 71.3, 71.9, 72.8 (C-2, C-3, C-4, C-5), 99.4 (C-1), 121.6, 128.7 (*C*H-arom), 134.9, 150.4 (C-arom), 169.4, 170.2, 170.7 (5x O*C*OCH_3 overlap). HRMS (TOF ESI$^+$): *m/z* calcd for $[C_{23}H_{28}O_{12}]Na^+$: 519.1479, found 519.1483; calcd for $[C_{23}H_{28}O_{12}]K^+$: 535.1218, found 535.1254.

4-Acetoxy-3-methoxybenzyl 2,3,4,6-tetra-*O*-acetyl-β-D-glucopyranoside (21b): White crystals, m. p. 116-117 ºC (*i*-propanol), $[\alpha]_D$ - 46.0 (*c* 1.0, $CHCl_3$). ^1H NMR (300 MHz, $CDCl_3$): δ = 2.00, 2.03, 2.04, 2.10, 2.31 (each s, 15 H, 5x OCOCH_3), 3.71 (ddd, 1 H, *J* = 2.5, 4.5, 9.5 Hz, H-5), 3.83 (s, 3 H, OCH_3), 4.16 (dd, 1 H, *J* = 2.2, 12.3 Hz, H-6a), 4.27 (dd, 1 H, *J* = 4.5, 12.3 Hz, H-6b), 4.57 and 4.87 (each d, 2x1 H, *J* = 12.3 Hz, CH_2), 4.60 (d, 1 H, *J* = 7.5 Hz, H-1), 5.05-5.22 (m, 3 H, H-2, H-3, H-4), 6.85 (d, 1 H, *J* = 8.1 Hz, H-arom), 6.93 (s, 1 H, H-arom), 7.00 (d, 1 H, *J* = 8.0 Hz, H-arom). ^{13}C NMR (75 MHz, $CDCl_3$): δ = 20.6 (5x OCO*C*H_3 overlap), 55.9 (OCH_3), 61.9 (C-6), 68.4 (CH_2), 70.4, 71.3, 71.9, 72.7 (C-2, C-3, C-4, C-5), 99.6 (C-1), 111.6, 119.5, 122.6 (CH-arom), 135.7, 139.3, 150.4 (C-arom), 169.0, 169.2, 169.3, 170.2, 170.6 (5x O*C*OCH_3). HRMS (TOF ESI^+): *m/z* calcd for $[C_{24}H_{30}O_{13}]Na^+$: 549.1584, found 549.1592; calcd for $[C_{24}H_{30}O_{13}]K^+$: 565.1324, found 565.1331.

4-Acetoxy-3,5-dimethoxybenzyl 2,3,4,6-tetra-*O*-acetyl-β-D-glucopyranoside (21c): White crystals, m. p. 123-124 ºC (*i*-propanol), $[\alpha]_D$ - 35.3 (*c* 1.0, $CHCl_3$). ^1H NMR (300 MHz, $CDCl_3$): δ = 2.00, 2.01, 2.03, 2.10, 2.33 (each s, 15 H, 5x OCOCH_3), 3.72 (ddd, 1 H, *J* = 2.4, 4.3, 9.4 Hz, H-5), 3.82 (s, 6 H, 2x OCH_3), 4.17 (dd, 1 H, *J* = 2.2, 12.3 Hz, H-6a), 4.28 (dd, 1 H, *J* = 4.5, 12.3 Hz, H-6b), 4.57 and 4.88 (each d, 2x1 H, *J* = 12.3 Hz, CH_2), 4.63 (d, 1 H, *J* = 7.7 Hz, H-1), 5.07-5.25 (m, 3 H, H-2, H-3, H-4), 6.56 (s, 2 H, H-arom). ^{13}C NMR (75 MHz, $CDCl_3$): δ = 20.4, 20.6, 20.7 (5x OCO*C*H_3 overlap), 56.1 (2x OCH_3), 61.9 (C-6), 68.4 (CH_2), 70.7, 71.3, 71.8, 72.7 (C-2, C-3, C-4, C-5), 99.7 (C-1), 103.8(2x) (CH-arom), 128.1 (C-arom), 135.3(2x) (C-arom), 152.1 (C-arom), 168.7, 169.2, 169.4, 170.2, 170.6 (5x O*C*OCH_3). HRMS (TOF ESI^+): *m/z* calcd for $[C_{25}H_{32}O_{14}]$ Na^+: 579.1690, found 579.1701.

4-Acetoxyphenetyl 2,3,4,6-tetra-*O*-acetyl-β-D-glucopyranoside (22): White crystals, m. p. 87-90 ºC (*i*-propanol), $[\alpha]_D$ - 28.8 (*c* 1.0, $CHCl_3$). ^1H NMR (400 MHz, $CDCl_3$) δ = 1.91, 1.99, 2.02, 2.09, 2.28 (each s, 15 H, 5x OCOCH_3), 2.82-2.93 (m, 2 H, CH_2), 3.59-3.73 (m, 2 H, 1H(CH_2), H-5), 4.10-4.16 (m, 1 H, 1H(CH_2)), 4.13 (dd, 1 H, *J* = 12.3, 2.2 Hz, H-6a), 4.26 (dd, 1 H, *J* = 12.3, 4.9 Hz, H-6b), 4.48 (d, 1 H, *J* = 7.9 Hz, H-1), 4.99 (dd, 1 H, *J* = 9.5, 7.9 Hz, H-

2), 5.08 (t, 1 H, *J* = 9.6 Hz, H-4), 5.17 (t, 1 H, *J* = 9.5 Hz, H-3), 6.98 (d, 2 H, *J* = 8.4 Hz, H-arom), 7.20 (d, 2 H, *J* = 8.5 Hz, H-arom). ^{13}C NMR (101 MHz, $CDCl_3$) δ = 20.6, 20.7, 21.1 (5x OCO*C*H_3 overlap), 35.3 (CH_2), 61.9 (C-6), 68.4 (C-4), 70.4 (CH_2), 71.1 (C-2), 71.8 (C-5), 72.7 (C-3), 100.7 (C-1), 121.3, 129.9 (4x CH-arom), 136.0, 149.1 (C-arom), 169.3(2x),169.5, 170.2, 170.6 (5x O*C*OCH_3). HRMS (TOF ESI$^+$): *m/z* calcd for $[C_{24}H_{30}O_{12}]Na^+$: 533.1635, found 533.1642.

4-Acetoxy-3-methoxycinnamyl 2,3,4,6-tetra-*O*-acetyl-β-D-glucopyranoside (23): White crystals, m. p. 87 - 90 ºC (*i*-propanol), $[\alpha]_D$ - 27.0 (*c* 1.0, $CHCl_3$). ^{1}H NMR (300 MHz, $CDCl_3$): δ = 2.01, 2.03, 2.06, 2.08, 2.31 (each s, 15 H, 5x OCO*C*H_3), 3.71 (ddd, 1 H, *J* = 2.5, 4.7, 9.8 Hz, H-5), 3.85 (s, 3 H, OCH_3), 4.17 (dd, 1 H, *J* = 2.3, 12.3 Hz, H-6a), 4.27 (dd, 1 H, *J* = 4.7, 12.3 Hz, H-6b), 4.27 and 4.49 (dd and ddd, 2x1 H, *J* = 1.4, 5.3, 6.2, 12.7 Hz, CH_2), 4.62 (d, 1 H, *J* = 7.9 Hz, H-1), 5.02-5.22 (m, 3 H, H-2, H-3, H-4), 6.17 (ddd, 1 H, *J* = 5.6, 6.3, 15.9 Hz, H-A), 6.56 (d, 1 H, *J* = 15.9 Hz, H-B), 6.91-7.00 (m, 3 H, H-arom).^{13}C NMR (75 MHz, $CDCl_3$): δ = 20.6, 20.7 (5x OCO*C*H_3 overlap), 55.8 (OCH_3), 61.9 (C-6), 69.7 (CH_2), 68.4, 71.3, 71.8, 72.8 (C-2, C-3, C-4, C-5), 99.7 (C-1), 110.2, 119.2, 122.8 (CH-arom), 124.9 (C-A), 132.3 (C-B), 135.4, 139.5, 151.1 (C-arom), 169.0, 169.4(2x), 170.3, 170.7 (5x O*C*OCH_3 overlap). HRMS (TOF ESI$^+$): *m/z* calcd for $[C_{26}H_{32}O_{13}]Na^+$: 575.1741, found 575.1759.

4-Acetoxy-3-methoxycinnamaldehyde (24): Colourless oil. ^{1}H NMR (300 MHz, $CDCl_3$): δ = 2.33 (s, 3 H, $OCOCH_3$), 3.88 (s, 3 H, OCH_3), 6.67 (dd, 1 H, *J* = 7.6, 15.9 Hz, H-A), 7.08-7.18 (m, 3 H, H-arom), 7.44 (d, 1 H, *J* = 15.9 Hz, H-B), 9.70 (d, 1 H, *J* = 7.6 Hz, CH=O). ^{13}C NMR (75 MHz, $CDCl_3$): δ = 20.6 (O*C*OCH_3), 55.9 (OCH_3), 111.4, 121.8, 123.5 (CH-arom), 128.7 (CH-A), 132.9, 142.2, 151.6 (C-arom), 151.8 (CH-B), 168.6 (O*C*OCH3), 193.4 (CH=O).

4-Acetoxy-3-methoxybenzyl 2,3,4,6-tetra-*O*-acetyl-β-D-galactopyranoside (25): Colourless oil, $[\alpha]_D$ - 24.0 (*c* 1.0, $CHCl_3$). ^{1}H NMR (300 MHz, $CDCl_3$): δ = 1.98, 2.00, 2.06, 2.16, 2.31 (each s, 15 H, 5x $OCOCH_3$), 3.84 (s, 3 H, OCH_3), 3.89 (m, 1 H, H-5), 4.16 (dd, 1 H, *J* = 6.8, 11.3 Hz, H-6a), 4.21 (dd, 1 H, *J* = 6.6, 11.2 Hz, H-6b), 4.56 (d, 1H, *J* = 7.8 Hz, H-

1), 4.60 and 4.90 (each d, 2x H, *J* = 12.3 Hz, CH_2), 5.00 (dd, 1 H, *J* = 3.4, 10.5 Hz, H-3), 5.29 (dd, 1 H, *J* = 8.0, 10.5 Hz, H-2), 5.40 (bd, 1 H, *J* = cca 1.0, 2,6 Hz, H-4), 6.85 (dd, 1 H, *J* = 1.4, 8.1 Hz, H-arom), 6.94 (d, 1 H, *J* = 1.4 Hz, H-arom), 7.00 (d, 1 H, *J* = 8.0 Hz, H-arom). ^{13}C NMR (75 MHz, $CDCl_3$): δ = 20.6 (5x OCO*C*H_3 overlap), 55.8 (OCH_3), 61.3 (C-6), 70.4 (CH_2), 67.0, 68.8, 70.7, 70.8 (C-2, C-3, C-4, C-5), 100.1 (C-1), 111.6, 119.5, 122.6 (3x CH-arom), 135.8, 139.3, 151.1 (3x C-arom), 169.0, 169.4, 170.1, 170.2, 170.3 (5x O*C*OCH_3). HRMS (TOF ESI$^+$): *m/z* calcd for $[C_{24}H_{30}O_{13}]Na^+$: 549.1584, found 549.1560; calcd for $[C_{24}H_{30}O_{13}]$ K^+: 565.1324, found 565.1357.

4-Acetoxy-3-methoxybenzyl 2,3,4,6-tetra-*O*-acetyl-α-D-mannopyranoside (26):

Colourless oil, $[\alpha]_D$ + 51.0 (*c* 1.0, $CHCl_3$). ^{1}H NMR (300 MHz, $CDCl_3$): δ = 1.99, 2.04, 2.11, 2.15, 2.31 (each s, 15 H, 5x $OCOCH_3$), 3.86 (s, 3 H, OCH_3), 4.01 (ddd, 1 H, *J* = 2.5, 4.6, 9.7 Hz, H-5), 4.09 (dd, 1 H, *J* = 2.4, 12.2 Hz, H-6a), 4.29 (dd, 1 H, *J* = 5.1, 12.2 Hz, H-6b), 4.55 and 4.69 (each d, 2x1 H, *J* = 12.1 Hz, CH_2), 4.89 (d, 1 H, *J* < 1 Hz, H-1), 5.27-5.41 (m, 3 H, H-2, H-3, H-4), 6.90 (dd, 1 H, *J* = 1.8, 8.0 Hz, H-arom), 6.97 (d, 1 H, *J* = 1.6 Hz, H-arom), 7.02 (d, 1 H, *J* = 8.0 Hz, H-arom). ^{13}C NMR (75 MHz, $CDCl_3$): δ = 20.7, 20.9 (5x OCO*C*H_3 overlap), 55.9 (OCH_3), 62.4 (C-6), 66.1, 68.8, 69.1, 69.5 (C-2, C-3, C-4, C-5), 69.1 (CH_2), 96.5 (C-1, $^1J_{C1,H1}$ 170.3 Hz), 112.0, 120.2, 122.8 (3x CH-arom), 135.1, 139.6, 151.2 (3x C-arom), 169.0, 169.7, 169.9, 170.0, 170.6 (5x O*C*OCH_3). HRMS (TOF ESI$^+$): *m/z* calcd for $[C_{24}H_{30}O_{13}]Na^+$: 549.1584, found 549.1562; calcd for $[C_{24}H_{30}O_{13}]K^+$: 565.1324, found 565.1347.

Methyl 4-acetoxy-3-methoxybenzyl 2,3,4-tri-*O*-acetyl-β-D-glucopyranuronate (27)

Colourless oil, $[\alpha]_D$ - 51.0 (*c* 1.0, $CHCl_3$). ^{1}H NMR (300 MHz, $CDCl_3$): δ = 1.99, 2.01, 2.02, 2.31 (each s, 12 H, 4x $OCOCH_3$), 3.76 (s, 3 H, CO_2CH_3), 3.83 (s, 3 H, OCH_3), 4.05 (d, 1 H, *J* = 9.3 Hz, H-5), 4.59 (d, 1 H, *J* = 12.3 Hz, CH_2), 4.64 (d, 1 H, *J* = 7.5 Hz, H-1), 4.91 (d, 1 H, *J* = 12.3 Hz, CH_2), 5.06-5.12 (m, 1 H, H-2), 5.24-5.27 (m, 2 H, H-3, H-4), 6.84 (dd, 1 H, *J* = 1.6, 8.0 Hz, H-arom), 6.94 (d, 1 H, *J* = 1.4 Hz, H-arom), 6.99 (d, 1 H, *J* = 8.0 Hz, H-arom). ^{13}C NMR (75 MHz, $CDCl_3$): δ = 20.5, 20.6 (4x OCO*C*H_3 overlap), 52.9 (CO_2*C*H_3), 55.9 (OCH_3),

69.3, 70.6, 71.2, 72.0, 72.6 (C-2, C-3, C-4, C-5, CH_2), 99.5 (C-1), 111.7, 119.6, 122.6 (3x CH-arom), 135.5, 139.4, 151.1 (3x C-arom), 167.2 (*C*OOCH$_3$), 169.0, 169.1, 169.3, 170.1 (4x O*C*OCH$_3$). HRMS (TOF ESI$^+$): *m/z* calcd for $[C_{23}H_{28}O_{13}]Na^+$: 535.1428, found 535.1440; calcd for $[C_{23}H_{28}O_{13}]K^+$: 551.1167, found 551.1172.

4-Acetoxy-3-methoxybenzyl (2,3,4,6-tetra-*O*-acetyl-β-D-galactopyranosyl)-(1→4)-2,3,6-tri-*O*-acetyl-β-D-glucopyranoside (28): White foam, $[\alpha]_D$ - 23.0 (*c* 1.0, $CHCl_3$). ^{1}H NMR (300 MHz, $CDCl_3$): δ = 1.97, 2.00, 2.05(2x), 2.06, 2.13, 2.15, 2.31 (each s, 24 H, 8x $OCOCH_3$ overlap), 3.61 (m, 1H, H-5), 3.82 (s, 3 H, OCH_3), 3.80-3.90 (m, 2 H, H-4, H-5'), 4.05-4.17 (m, 3 H, H-6a, H-6'a, H-6'b), 4.49 (d, 1 H, *J* = 7.8 Hz, H-1'), 4.56 and 4.84 (each d, 2x 1 H, *J* = 12.4 Hz, CH_2), 4.52 (m, 1 H, H-6b), 4.57 (d, 1 H, *J* = 7.9 Hz, H-1), 4.96 (dd, 1 H, *J* = 3.4, 10.4 Hz, H-3'), 4.98 (t, 1 H, *J* = 7.9, 8.6 Hz, H-2), 5.11 (dd, 1 H, *J* = 7.9, 10.4 Hz, H-2'), 5.19 (t, 1 H, *J* = 9.2, 9.3 Hz, H-3), 5.35 (bd, 1 H, *J* = 2.8 Hz, H-4'), 6.84 (d, 1 H, *J* = 8.1 Hz, H-arom), 6.91 (s, 1 H, H-arom), 6.99 (d, 1 H, *J* = 8.0 Hz, H-arom). ^{13}C NMR (75 MHz, $CDCl_3$): δ = 20.6, 20.8 (8x OCO*C*H$_3$ overlap), 55.8 (OCH_3), 60.8, 61.9 (C-6, C-6'), 70.4 (CH_2), 66.6, 69.1, 70.7, 70.9, 71.7, 72.7(2x), 76.1 (C-2, C-3, C-4, C-5, C-2', C-3', C-4', C-5', overlap), 99.4 (C-1'), 101.0 (C-1), 111.5, 119.5, 122.6 (3x CH-arom), 128.2, 129.0, 151.1 (3x C-arom), 169.0, 169.6, 169.7, 170.1, 170.3 (8x O*C*OCH$_3$ overlap). HRMS (TOF ESI$^+$): *m/z* calcd for $[C_{36}H_{46}O_{21}]H^+$: 815.2610, found 815.2623; calcd for $[C_{36}H_{46}O_{21}]Na^+$: 837.2429, found 837.2418.

4-Acetoxy-3-methoxybenzyl 2,3,5-tri-*O*-acetyl-α-L-arabinofuranoside (29): Colourless oil, $[\alpha]_D$ - 38.0 (*c* 1.0, $CHCl_3$). ^{1}H NMR (300 MHz, $CDCl_3$): δ = 2.09, 2.10, 2.11, 2.31 (each s, 12 H, 4x $OCOCH_3$), 3.85 (s, 3 H, OCH_3), 4.28 (m, 2 H, H-4, H-5a), 4.43 (dd, 1 H, *J* = 2.6, 11.0 Hz, H-5b), 4.56 and 4.75 (each d, 2x 1 H, *J* = 12.4 Hz, CH_2), 5.02 (dd, 1 H, *J* = 1.2, 5.1 Hz, H-3), 5.09 (bs, 1 H, H-1), 5.17 (d, 1 H, *J* = 1.4 Hz, H-2), 6.91 (d, 1 H, *J* = 8.0 Hz, H-arom), 6.99 (s, 1 H, H-arom), 7.00 (d, 1 H, *J* = 7.9 Hz, H-arom).^{13}C NMR (75 MHz, $CDCl_3$): δ = 20.6, 20.7 (4x OCO*C*H$_3$ overlap), 55.8 (OCH_3), 63.2 (C-4, C-5, overlap), 68.3 (CH_2), 80.5, 81.3 (C-2, C-3), 104.6 (C-1), 111.6, 119.8, 122.6 (3x CH-arom), 136.0, 139.2, 151.1 (3x C-arom),

169.0, 169.6, 170.1, 170.6 (4x O*C*OCH$_3$). HRMS (TOF ESI$^+$): *m/z* calcd for [$C_{21}H_{26}O_{11}$]Na^+: 477.1373, found 477.1395; calcd for [$C_{21}H_{26}O_{11}$]K^+: 493.1112, found 493.1144.

4-Acetoxy-3-methoxybenzyl 2,3,4-tri-*O*-acetyl-β-D-xylopyranoside (30): White crystals, m. p. 125-126 ºC (*i*-propanol), $[\alpha]_D$ - 75.9 (*c* 1.0, $CHCl_3$). ^{1}H NMR (300 MHz, $CDCl_3$): δ = 2.02, 2.03, 2.05, 2.31 (each s, 12 H, 4x $OCOCH_3$), 3.39 (dd, 1 H, *J* = 8.5, 11.9 Hz, H-5a), 3.83 (s, 3 H, OCH_3), 4.16 (dd, 1 H, J = 5.0, 11.9 Hz, H-5b), 4.56 and 4.83 (each d, 2x1 H, *J* = 12.3 Hz, CH_2), 4.58 (d, 1 H, *J* = 6.5 Hz, H-1), 4.93-5.03 (m, 2 H, H-2, H-4), 5.16 (t, 1 H, *J* = 8.4 Hz, H-3), 6.86 (d, 1 H, *J* = 8.0 Hz, H-arom), 6.92 (s, 1 H, H-arom), 6.99 (d, 1 H, *J* = 8.0 Hz, H-arom). ^{13}C NMR (75 MHz, $CDCl_3$): δ = 20.7 (4x OCO*C*H$_3$ overlap), 55.9 (OCH_3), 62.0 (C-5), 70.0 (CH_2), 68.9, 70.7, 71.3 (C-2, C-3, C-4), 99.5 (C-1), 111.6, 119.6, 122.6 (3x CH-arom), 135.9, 139.3, 151.1 (3x C-arom), 169.0, 169.3, 169.8, 170.0 (4x O*C*OCH$_3$). HRMS (TOF ESI$^+$): *m/z* calcd for [$C_{21}H_{26}O_{11}$]Na^+:477.1373, found 477.1358.

General procedure for deprotection: Glycoside **21a–c, 22, 23** and **25–30** was dissolved in dry MeOH (9 mL), cooled to 4 °C and 0.5 M MeONa (1 mL) was added dropwise. The mixture was stirred at 4 °C until TLC (EtOAc/MeOH 6:1) showed that the reaction is complete. The pH of the mixture was brought pH 5 using Amberlite IR 120 (H^+ form). The resin was filtered off, the filtrate concentrated and the residue was subjected to column chromatography (EtOAc/MeOH 1:0→6:1).

4-Hydroxybenzyl β-D-glucopyranoside (31a): White foam, yield 86%, $[\alpha]_D$ - 46.4º (*c* 1.0, MeOH). ^{1}H NMR (300 MHz, CD_3OD): δ = 3.22-3.33 (m, 4 H, H-2, H-3, H-4, H-5), 3.67 (dd, 1 H, *J* = 5.4, 11.9 Hz, H-6b), 3.88 (dd, 1 H, *J* = 1.9, 11.9 Hz, H-6a), 4.31 (d, 1 H, *J* = 7.7 Hz, H-1), 4.55 and 4.79 (each d, 2x 1H, *J* = 11.2 Hz, CH_2), 6.75 (d, 2 H, *J* = 8.4 Hz, H-arom), 7.23 (d, 2 H, *J* = 8.4 Hz, H-arom).^{13}C NMR (75 MHz, CD_3OD): δ = 62.9 (C-6), 71.7 (CH_2), 71.8, 75.2, 78.0, 78.2 (C-2, C-3, C-4, C-5), 102.9 (C-1), 116.0(2x) (CH-arom), 129.7 (C-arom), 131.2(2x) (CH-arom), 158.4 (C-arom). HRMS (TOF ESI$^+$): *m/z* calcd for [$C_{13}H_{18}O_7$]Na^+: 309.0950, found 309.1007; calcd for [$C_{13}H_{18}O_7$]K^+: 325.0690, found 325.0759.

4-Hydroxy-3-methoxybenzyl β-D-glucopyranoside (1): White foam, yield 84%, $[\alpha]_D$ - 50.8 (*c* 1.0, MeOH). ^{1}H NMR (300 MHz, CD_3OD): δ = 3.21-3.35 (m, 4 H, H-2, H-3, H-4,H-5), 3.68 (dd, 1 H, *J* = 5.1, 11.9 Hz, H-6b), 3.85 (s, 3 H, OCH_3), 3.89 (bd, 1 H, *J* = cca 1, 12.0 Hz, H-6a), 4.32 (d, 1 H, *J* = 7.6 Hz, H-1), 4.58 and 4.81 (each d, 2x 1 H, *J* = 11.4 Hz, CH_2), 6.74 (d, 1 H, *J* = 8.0 Hz, H-arom), 6.83 (d, 1 H, *J* = 8.0 Hz, H-arom), 7.05 (s, 1 H, H-arom). ^{13}C NMR (75 MHz, CD_3OD): δ = 56.4 (OCH_3), 62.9 (C-6), 71.8 (CH_2), 71.8, 75.2, 78.1, 78.2 (C-2, C-3, C-4, C-5), 102.8 (C-1), 113.4, 115.8, 122.5 (CH-arom), 130.4, 147.4, 149.0 (C-arom). HRMS (TOF ESI$^+$): *m/z* calcd for $[C_{14}H_{20}O_8]Na^+$: 339.1056, found 339.1064.

4-Hydroxy-3,5-dimethoxybenzyl β-D-glucopyranoside (31b): White foam, yield 81%, $[\alpha]_D$ - 38.3 (*c* 1.0, MeOH). ^{1}H NMR (300 MHz, CD_3OD): δ = 3.29-3.41 (m, 4 H, H-2, H-3, H-4, H-5), 3.74 (dd, 1 H, *J* = 5.3, 11.8 Hz, H-6b), 3.88 (s, 6 H, 2x OCH_3), 3.94 (bd, 1 H, *J* = cca 1 Hz, cca 12 Hz, H-6a), 4.36 (d, 1 H, *J* = 7.6 Hz, H-1), 4.64 and 4.85 (each d, 2x 1 H, *J* = 11.7 Hz, CH_2), 6.77 (s, 2 H, H-arom). ^{13}C NMR (75 MHz, CD_3OD): δ = 56.8(2x) (OCH_3), 62.9 (C-6), 71.8 (CH_2), 71.9, 75.1, 78.1(2x) (C-2, C-3, C-4, C-5), 102.7 (C-1), 106.8(2x) (CH-arom), 129.5, 136.1, 149.1(2x) (C-arom). HRMS (TOF ESI$^+$): *m/z* calcd for $[C_{15}H_{22}O_9]Na^+$: 369.1162, found 369.1168.

4-Hydroxyphenetyl β-D-glucopyranoside (salidroside, 2): White crystals, yield 90%, m. p. 160-162 ºC (MeOH /$CHCl_3$), 163-165.5 °C (hexane/EtOH) [7], 158-159 °C, n-hexane/MeOH) [8], 159-160 °C [9,10]; $[\alpha]_D$ - 31.0 (*c* 1.0, CH_3OH), $[\alpha]_D^{23}$ - 40.6 (c 0.65, H_2O) [7], $[\alpha]_D^{26}$ - 28.4 (c 0.5, MeOH) [9,10]. ^{1}H NMR (400 MHz, CD_3OD): δ = 2.83 (ddd, 2 H, *J* = 8.5, 6.9, 2.1 Hz, CH_2), 3.18 (dd, 1 H, *J* = 9.0, 7.7 Hz, H-2), 3.22-3.30 (m, 2 H, H-4, H-5), 3.35 (t, 1 H, *J* = 9.0, 9.0 Hz, H-3), 3.59-3.75 (m, 2 H, 1H(C*H*$_2$), H-6b), 3.86 (dd, 1 H, *J* = 11.8, 1.9 Hz, H-6a), 4.03 (ddd, 1 H, *J* = 9.6, 8.1, 7.0 Hz, 1H(CH_2)), 4.29 (d, 1 H, *J* = 7.8 Hz, H-1), 6.69 (d, 2 H, *J* = 8.5 Hz, H-arom), 7.06 (d, 2 H, *J* = 8.5 Hz, H-arom).^{13}C NMR (101 MHz, CD_3OD): δ = 36.4 (CH_2), 62.7 (C-6), 71.6 (C-4), 72.1 (CH_2), 75.1 (C-2), 77.9 (C-5), 78.1 (C-3), 104.4 (C-1), 116.1,

130.9 (CH-arom), 130.7, 156.8 (C-arom). HRMS (TOF ESI$^+$): *m/z* calcd for $[C_{14}H_{20}O_7]Na^+$: 323.1107, found 323.1110.

4-Hydroxy-3-methoxycinnamyl β-D-glucopyranoside (citrusin D, coniferin, 3): A pale yellow foam, yield 88%, $[\alpha]_D$ - 33.0 (*c* 1.0, MeOH), $[\alpha]_D^{25}$ - 47.7 (c = 1.0, MeOH) [11], $[\alpha]_D^{23}$ - 36.0 (c 0.54, MeOH) [9,12]. ^{1}H NMR (300 MHz, CD_3OD): δ = 3.20-3.36 (m, 4 H, H-2, H-3, H-4, H-5), 3.67 (dd, 1 H, *J* = 4.6, 11.8 Hz, H-6b), 3.86 (s, 3 H, OCH_3), 3.83-3.93 (m, 1 H, H-6a), 4.37 (d, 1 H, *J* = 7.7 Hz, H-1), 4.29 and 4.49 (dd and dd, 2x1 H, *J* = 5.8, 6.9, 12.5 Hz, CH_2), 6.19 (td, 1 H, *J* = 6.4, 15.9 Hz, H-A), 6.58 (d, 1 H, *J* = 16.0 Hz, H-B), 6.73 (d, 1 H, *J* = 8.1Hz, H-arom), 6.86 (bd, 1 H, *J* = 8.0 Hz, H-arom), 7.01 (bs, 1 H, H-arom). ^{13}C NMR (75 MHz, CD_3OD): δ = 56.4 (OCH_3), 62.8 (C-6), 71.1 (CH_2), 71.7, 75.2, 77.9, 78.2 (C-2, C-3, C-4, C-5), 103.2 (C-1), 110.6, 116.2, 121.2 (CH-arom), 123.8 (C-A), 134.3 (C-B), 130.4, 147.7, 149.1 (C-arom). HRMS (TOF ESI$^+$): *m/z* calcd for $[C_{15}H_{20}O_8]Na^+$: 351.1056, found 351.1068.

4-Hydroxy-3-methoxybenzyl β-D-galactopyranoside (32): White foam, yield 83%, $[\alpha]_D$ - 29.9 (*c* 1.0, MeOH). ^{1}H NMR (300 MHz, CD_3OD): δ = 3.44-3.53 (m, 3 H, H-2, H-3, H-5), 3.74-3.82 (m, 3 H, H-4, H-6a, H-6b), 3.85 (s, 3 H, OCH_3), 4.27 (d, 1 H, *J* = 7.7 Hz, H-1), 4.58 and 4.80 (each d, 2x 1 H, *J* = 11.4 Hz, CH_2), 6.74 (d, 1 H, *J* = 8.0 Hz, H-arom), 6.82 (dd, 1H, *J* = 1.6, 8.0 Hz, H-arom), 7.05 (d, 1H, *J* = 1.5 Hz, H-arom).^{13}C NMR (75 MHz, CD_3OD): δ = 56.4 (OCH_3), 62.7 (C-6), 71.7 (CH_2), 70.4, 72.6, 75.1, 76.8 (C-2, C-3, C-4, C-5), 103.4 (C-1), 113.4, 115.8, 122.5 (CH-arom), 130.5, 147.4, 149.0 (C-arom). HRMS (TOF ESI$^+$): *m/z* calcd for $[C_{14}H_{20}O_8]Na^+$: 339.1056, found 339.1059; calcd for $[C_{14}H_{20}O_8]K^+$: 355.0795, found 355.0799.

4-Hydroxy-3-methoxybenzyl α-D-mannopyranoside (33): White foam, yield 85%, $[\alpha]_D$ + 70.1 (*c* 1.0, MeOH). ^{1}H NMR (300 MHz, CD_3OD): δ = 3.59-3.86 (m, 6 H, H-2, H-3, H-4, H-5, H-6a, H-6b), 3.84 (s, 3 H, OCH_3), 4.43 and 4.63 (each d, 2x 1H, *J* = 11.4 Hz, CH_2), 4.81 (d, 1 H, *J* = 1.4 Hz, H-1), 6.76 (d, 1 H, *J* = 8.0 Hz, H-arom), 6.81 (dd, 1H, *J* = 1.6, 8.0 Hz, H-arom), 6.93 (d, 1H, *J* = 1.4 Hz, H-arom). ^{13}C NMR (75 MHz, CD_3OD): δ = 56.4 (OCH_3), 63.0 (C-6),

70.0 (CH_2), 68.7, 72.3, 72.7, 74.8 (C-2, C-3, C-4, C-5), 100.3 (C-1, $^1J_{C,H}$ = 170.3 Hz), 113.3, 116.0, 122.6 (CH-arom), 130.4, 147.4, 149.0 (C-arom). HRMS (TOF ESI$^+$): *m/z* calcd for $[C_{14}H_{20}O_8]Na^+$: 339.1056, found 339.1063; calcd for $[C_{14}H_{20}O_8]K^+$: 355.0795, found 355.0810.

Methyl 4-acetoxy-3-methoxybenzyl β-D-glucopyranuronate (34): White foam, yield 85%, $[\alpha]_D$ - 57 (*c* 1.0, MeOH). ^{1}H NMR (300 MHz, CD_3OD): δ = 3.25-3.40 (m, 2 H, H-2, H-3), 3.53 (t, 1 H, *J* = 9.2 Hz, H-4), 3.78 (s, 3 H, CO_2CH_3), 3.80 (d, 1 H, *J* = 8.4 Hz, H-5), 3.84 (s, 3 H, OCH_3), 4.38 (d, 1 H, *J* = 7.6 Hz, H-1), 4.52 and 4.75 (each d, 2x 1 H, *J* = 11.5 Hz, CH_2), 6.72-6,81 (m, 2 H, H-arom), 7.01 (bs, 1 H, H-arom),.^{13}C NMR (75 MHz, CD_3OD): δ = 52.9 ($CO_2\mathit{C}H_3$), 56.4 (OCH_3), 72.3, 73.2, 74.7, 76.8, 77.3 (C-2, C-3, C-4, C-5, CH_2), 103.4 (C-1), 113.3, 115.8, 122.4 (CH-arom), 130.0, 147.4, 148.9 (C-arom), 171.3 (*C*$OOCH_3$). HRMS (TOF ESI$^+$): *m/z* calcd for $[C_{15}H_{20}O_9]Na^+$: 367.1005, found 367.1019; calcd for $[C_{15}H_{20}O_9]K^+$: 383.0744, found 383.0749.

4-Hydroxy-3-methoxybenzyl 4-O-β-D-galactopyranosyl-β-D-glucopyranoside (35): White foam, yield 84%, $[\alpha]_D$ - 7.0 (*c* 1.0, H_2O). ^{1}H NMR (300 MHz, D_2O): δ = 3.52-3.94 (m, 12 H, H-2, H-3, H-4, H-6a, H-6b, H-2', H-3', H-4',H-6'a, H-6'b), 3.85 (s, 3 H, OCH_3), 4.41 and 4.48 (each d, 2x 1 H, *J* = 7.7 Hz, and *J* = 8.0 Hz, H-1 and H-1'), 4.64 and 4.81 (each d, 2x 1 H, *J* = 11.4 Hz, CH_2), 6.88-6.91 (m, 2 H, H-arom), 7.10 (bs, 1 H, H-arom). ^{13}C NMR (75 MHz, D_2O): δ = 56.8 (OCH_3), 61.1, 61.9 (C-6, C-6'), 72.3 (CH_2), 69.5, 71.9, 73.5, 73.7, 75.4, 75.7, 76.3, 79.4 (C-2, C-3, C-4, C-5, C-2', C-3', C-4', C-5'), 101.7, 103.8 (C-1, C-1'), 114.1, 116.2, 123.1 (*C*H-arom), 130.0, 145.8, 148.3 (C-arom). HRMS (TOF ESI$^+$): *m/z* calcd for $[C_{20}H_{30}O_{13}]Na^+$: 501.1584, found 501.1597; calcd for $[C_{20}H_{30}O_{13}]K^+$: 517.1324, found 517.1337.

4-Hydroxy-3-methoxybenzyl α-L-arabinofuranoside (36): Colourless oil, yield 80%, $[\alpha]_D$ - 34.6 (c 1.0, MeOH). ^{1}H NMR (300 MHz, CD_3OD): δ = 3.66 (dd, 1 H, *J* = 5.4, 11.9 Hz, H-5b), 3.79 (dd, 1 H, *J* = 3.1, 11.9 Hz, H-5a), 3.81-3.88 (m, 2 H, H-3, H-4), 3.85 (s, 3 H, OCH_3), 3.97 (bd, 1 H, *J* = 3.2 Hz, H-2), 4.43 and 4.65 (each d, 2x 1 H, *J* = 11.5 Hz, CH_2), 4.93 (d, 1 H, *J* = 1.3 Hz, H-1), 6.74 (d, 1 H, *J* = 7.9 Hz, H-arom), 6.79 (dd, 1H, *J* = 1.6, 8.0 Hz, H-arom), 6.95

(d, 1H, J = 1.6 Hz, H-arom). ^{13}C NMR (75 MHz, CD_3OD): δ = 56.4 (OCH_3), 63.1 (C-5), 70.2 (CH_2), 79.0, 83.7, 85.6 (C-2, C-3, C-4), 108.2 (C-1), 113.2, 115.9, 122.4 (CH-arom), 130.6, 147.4, 149.0 (C-arom). HRMS (TOF ESI$^+$): *m/z* calcd for $[C_{13}H_{18}O_7]Na^+$: 309.0950, found 309.1000.

4-Hydroxy-3-methoxybenzyl β-D-xylopyranoside (37): White foam, yield 88%, $[\alpha]_D$ - 71.0 (*c* 1.0, MeOH). ^{1}H NMR (300 MHz, CD_3OD): δ = 3.18 (dd, 1 H, J = 10.4, 11.3 Hz, H-5a), 3.21-3.35 (m, 2 H, H-2, H-3), 3.48 (ddd, 1 H, J = 5.4, 8.5, 10.1, H-4), 3.84 (s, 3 H, OCH_3), 3.89 (dd, 1 H, J = 5.3, 11.4 Hz, H-5b), 4.27 (d, 1 H, J = 7.3 Hz, H-1), 4.52 and 4.74 (each d, 2x 1 H, J = 11.4 Hz, CH_2), 6.74 (d, 1 H, J = 8.0 Hz, H-arom), 6.80 (dd, 1 H, J = 1.6, 8.0 Hz, H-arom), 7.01 (d, 1 H, J = 1.5 Hz, H-arom). ^{13}C NMR (75 MHz, CD_3OD): δ = 56.4 (OCH_3), 67.0 (C-5), 71.9 (CH_2), 71.3, 75.0, 77.9 (C-2, C-3, C-4), 103.7 (C-1), 113.3, 115.9, 122.4 (CH-arom), 130.3, 147.4, 149.1 (C-arom). HRMS (TOF ESI$^+$): *m/z* calcd for $[C_{13}H_{18}O_7]Na^+$: 309.0950, found 309.0977.

References

1. Lee, J.; Ryu, T.; Park, S.; Lee, P. H. *J. Org. Chem.* **2012**, 77, 4821-4825.
2. Ciaramitaro, D.; Steelink, C. *Phytochemistry* **1975**, *14*, 543 – 552.
3. Mitchell, S. A.; Pratt, M. R.; Hruby, V. J.; Polt, R. *J. Org. Chem*. **2001**, *66*, 2327-2342.
4. Wenger, J.; Ley, S. V.; Kirschning, A.; Hansen, A-L.; Montenergo, G. J.; Baxendale, I. R. *Org. Lett.* **2012**, *14*, 696-699.
5. Jongkees, S. A. K.; Withers S. G. *J. Am. Chem. Soc*. **2011**, *133*, 19334-19337.
6. Zhang, Q.; Liu, H. *Bioorg. Med. Chem. Lett.* **2001**, *11*, 145-149.
7. Kuwajima, H.; Takai, Y.; Takaishi, K.; Inoue, K. *Chem. Pharm. Bull*. **1998**, *46*, 581-586.
8. Shi, T.; Chen, H.; Jing, L.; Liu, X.; Sun, X.; Jiang, R. *Synth. Commun*. **2011**, *41*, 2594-2600.
9. Akita, H.; Kawahara, E.; Kishida, M.; Kato, K. *J. Mol. Catal. B–Enzym.* **2006**, *40*, 8-15.

10. Akita, H.; Kurashima, K.; Nakamura, T.; Kato, K. *Tetrahedron–Asymmetr.* **1999**, *10*, 2429-2439.

11. Takano, T.; Tobimatsu,, Y.; Hosoya, T.; Hattori, T.; Ohnishi, J.; Takano, M.; Kamitakahara, H.; Nakatsubo, F. *J. Wood Chem. Technol*. **2006**, *26*, 215-229.

12. Kishida, M.; Akita, H. *Tetrahedron* **2005**, *61*, 10559-10568.
